# Antisense oligonucleotide inhibition of cholesteryl ester transfer protein enhances RCT in hyperlipidemic, CETP transgenic, LDLr^−/−^ mice

**DOI:** 10.1194/jlr.M036509

**Published:** 2013-10

**Authors:** Thomas A. Bell, Mark J. Graham, Richard G. Lee, Adam E. Mullick, Wuxia Fu, Dan Norris, Rosanne M. Crooke

**Affiliations:** Cardiovascular Antisense Drug Discovery Group, Isis Pharmaceuticals, Inc., Carlsbad, CA 92010

**Keywords:** cholesteryl ester transfer protein, cardiovascular disease, low density lipoprotein, lipoprotein metabolism

## Abstract

Due to their ability to promote positive effects across all of the lipoprotein classes, cholesteryl ester transfer protein (CETP) inhibitors are currently being developed as therapeutic agents for cardiovascular disease. In these studies, we compared an antisense oligonucleotide (ASO) inhibitor of CETP to the CETP small molecule inhibitor anacetrapib. In hyperlipidemic CETP transgenic (tg) mice, both drugs provided comparable reductions in total plasma cholesterol, decreases in CETP activity, and increases in HDL cholesterol. However, only mice treated with the antisense inhibitor showed an enhanced effect on macrophage reverse cholesterol transport, presumably due to differences in HDL apolipoprotein composition and decreases in plasma triglyceride. Additionally, the ASO-mediated reductions in CETP mRNA were associated with less accumulation of aortic cholesterol. These preliminary findings suggest that CETP ASOs may represent an alternative means to inhibit that target and to support their continued development as a treatment for cardiovascular disease in man.

Despite advances in diagnosis and treatment, cardiovascular disease (CVD) remains a leading cause of mortality in the United States (1). While statins and other lipid-lowering agents have provided potent lowering of proatherogenic LDL cholesterol (LDL-C) (2), cardiovascular mortality rates remain high, especially in patients with multiple risk factors. In recent years, low HDL cholesterol (HDL-C) levels have also been recognized by the National Cholesterol Education Program (NCEP) Adult Treatment Panel as an independent risk factor for CVD (3). Although the development of agents that increase levels of HDL-C has been challenging, cholesteryl ester transfer protein (CETP) has emerged as a potential therapeutic target.

In human plasma, CETP facilitates the movement of neutral lipid between lipoproteins, resulting in a net exchange of triglyceride (TG) from VLDL for cholesteryl ester (CE) from HDL (4). The impact of CETP on HDL metabolism was initially revealed in a series of genetic studies of Japanese families in which subjects with elevated HDL levels were found to have mutations that resulted in CETP deficiency (5–7). However, subsequent studies examining the relationship between CETP deficiency and CVD have been mixed. The seven-year prospective data from Honolulu Heart Study found no significant relationship between heterozygous mutations of CETP and CVD (8). Furthermore, results from this study and others indicated that individuals with loss-of-function mutations in CETP who have moderate HDL-C levels could have an increased CVD risk (8, 9). However, a large-scale meta-analysis of 92 studies with 113,833 subjects found increases in HDL-C associated with reductions in CETP protein and activity were atheroprotective (10). These observations were upheld in a genome-wide association study from the Women's Genome Health Study in which single nucleotide polymorphisms in the CETP gene were associated with an increase in HDL-C and a lower risk of developing CVD (11). In composite, these recent large-scale studies indicate CETP is proatherogenic and support the development of drugs targeting CETP.

The clinical development of CETP inhibitors has also been controversial. For example, torcetrapib, a small molecule inhibitor (SMI) of CETP, demonstrated significant increases in HDL-C and apoA-I as well as reductions in LDL-C (12, 13). However a Phase III trial of this drug was halted due to increased adverse events in the active treatment group (14). These negative results were attributed to off-target effects of torcetrapib on aldosterone levels that led to increases in blood pressure (15). More recently, the Phase III outcome trial of dalcetrapib, another CETP SMI, was stopped due to futility (16). Dalcetrapib was reported to be unique in that it primarily affected HDL metabolism and did not lower atherogenic lipoprotein cholesterol levels (17, 18). Compared with other CETP SMIs, dalcetrapib was administered at much higher doses to achieve a pharmacological effect, leading some to speculate that dalcetrapib was simply a less effective inhibitor (19). Despite these setbacks, potent CETP SMIs such as anacetrapib and evacetrapib, drugs that do not possess obvious harmful side effects and exert positive effects across all lipoprotein subclasses (20, 21), still hold promise as beneficial therapeutic agents.

The purpose of these studies was to evaluate an antisense oligonucleotide (ASO) inhibitor of CETP and to compare and contrast its pharmacological effects to those of the anacetrapib. These agents employ dramatically different and nonoverlapping mechanisms of action. The SMIs are reported to bind and inactivate CETP associated with the HDL particle (22), whereas the CETP ASO specifically targets and degrades CETP mRNA, thus significantly lowering the amount of protein that is synthesized in the liver. This difference in mechanism of action (MOA) could have significant implications on pharmacology because the HDL-SMI complex could affect HDL function. In a series of studies conducted in transgenic (tg) mice, administration of the ASO inhibited CETP activity and increased plasma HDL-C levels comparable with anacetrapib and provided similar reductions in total plasma cholesterol levels. Additionally, mice treated with the CETP ASO displayed enhanced reverse cholesterol transport (RCT) and reductions in plasma TG, and ASO-mediated reductions in CETP mRNA were associated with less accumulation of aortic cholesterol. The results suggest that an ASO could produce a unique therapeutic profile, distinct from the current CETP drugs being evaluated in late-stage clinical trials.

## MATERIALS AND METHODS

### Antisense oligonucleotides

A series of uniform chimeric 20-mer phosphorothioate ASOs containing 2’-*O*-methoxyethyl (2’ MOE) groups at positions 1–5 and 15–20 targeted to human CETP and a control ASO were synthesized and purified on an automated DNA synthesizer using phosphoramidite chemistry as previously described (23) The sequences evaluated were as follows: CETP ASO-(5′-CAGCACTTTAATGCCAGTGG-3′) and a control ASO-ISIS 141923 (5′-CCTTCCCTGAAGGTTCCTCC-3′, with underlined indicating 2’MOE modified bases.

### Small molecule inhibitor

Anacetrapib was synthesized by Dalton Pharma Services (Toronto, ON) according to methods previously described (24).

### Mice and diets

The human CETP tg mice used in these studies were a gift from the laboratory of Linda Curtiss (The Scripps Research Institute, La Jolla, CA), and the generation of these mice has been described in detail (25). The CETP tg LDLr ^−/−^ mice were produced by breeding the CETP tg with mice lacking a functional LDL receptor. The resulting heterozygous mice were backcrossed, resulting in homozygous CETP tg LDLr ^−/−^ mice. Mice were housed 3–5 to a cage on a 12 h light-dark cycle for the duration of the studies. All procedures and protocols were approved by an institutional animal care and use committee. For each experiment, mice were switched from chow to a Western diet (Harland Teklad Diet 88137), consisting of 42% of calories as fat and 0.15% cholesterol, a week before baseline plasma samples were drawn by a retro-orbital bleed. Mice in the treatment groups were balanced according to baseline plasma lipids, body weight, and food intake. All CETP small molecule inhibitors were added to the diet according to the dose indicated.

### Plasma chemistry and lipoprotein analysis

Plasma lipid and transaminase concentrations were analyzed on an Olympus AU400e automated clinical chemistry analyzer (Melville, NY). HDL-C concentration was determined using the HDL Cholesterol E Kit from Wako Diagnostics (Richmond, VA). HDL compositional analyses were performed on HDL collected by sequential ultracentrifugation according to established protocols (26). Briefly, the density of 500 μl of pooled plasma from each treatment group was adjusted to 1.063 g/ml with KBr. The plasma samples were spun in an ultracentrifuge at 100,000 rpm for 5 h to concentrate VLDL and LDL particles. The VLDL and LDL particles were removed, and the solution density was readjusted to 1.21 g/ml. Then the samples were respun at 100,000 rpm for 6 h to concentrate HDL. HDL total cholesterol (TC), free cholesterol (FC), TG, and phospholipid (PL) were determined by enzymatic assays. Protein concentration was found by Fisher Scientific's BCA (Pittsburgh, PA) assay, and CE was calculated by TC − FC × 1.67. Additionally, the concentration of apoA-I protein associated with HDL was determined from plasma samples in which apoB-bound lipoproteins were precipitated by the addition of phosphotungstate and magnesium salt. After centrifugation, the supernatants were collected and assayed for apoA-I by a SULFO-TAG NHS-Ester based mouse apoA-I ELISA developed in-house with Meso Scale Diagnostics (Rockville, MD) according to previously described protocols (27). Lipoprotein cholesterol distribution was analyzed by HPLC according to previously described methods (28). Briefly, after 12 weeks of treatment, terminal plasma samples were collected from mice treated with either saline, control ASO, CETP ASO, or anacetrapib. Three pooled 100 μl plasma samples (three mice/pool) per treatment group were injected onto a Superose 6 column, and VLDL, LDL, and HDL were separated by gel-filtration chromatography at a flow rate of 0.375 ml/min. Fractions were collected every minute and assayed for cholesterol content.

### Radiolabeled human HDL_3_

Human HDL_3_ was labeled with ^3^H-cholesteryl hexadecyl ether according to previously published techniques (29). Briefly, 1 mCi of ^3^H-cholesteryl hexadecyl ether (CEth) in toluene was evaporated under N_2_ and resuspended in 50 µl of ethanol. The radioisotope was added drop-wise to isolated human HDL purchased from Intracel Resources (Frederick, MD). After the addition of 200 mg of heat-inactivated LPDS, the solution was incubated overnight at 37°C. The HDL_3_ was isolated and concentrated by ultracentrifugation, and the samples were dialyzed in three exchanges of PBS.

### CETP protein and activity analysis

CETP protein concentration was determined by ELISA (Alpco Diagnostics, Salem, NH) and CETP activity was measured using a fluorometric assay kit from Roar Biomedical (New York, NY). The CETP activity assay was adapted for use on CETP tg mouse plasma by evaluating enzymatic activity across a range of plasma dilutions over time. A 30 min incubation of 5 µl of plasma diluted 10-fold was found to be optimal to compare relative activity across treatment groups.

### Comparative pharmacology studies

Eight- to ten-week-old male CETP tg and CETP tg LDLr^−/−^ mice were administered by intraperitoneal injection either saline, control ASO (15 mg/kg/wk), CETP ASO (15, 5, or 1.5 mg/kg/wk) or dietary anacetrapib (100, 50, 10 mg/kg/day). Mice were maintained on diet and drug treatment for six weeks. Three weeks into the study, the mice were fasted for 4 h, and an intermediate plasma sample was taken and analyzed for plasma cholesterol and TG. After six weeks of treatment, the mice were fasted for 4 h, and a terminal plasma sample was taken via heart puncture along with a liver sample for further analysis.

### In vivo RCT assay

The macrophage-to-feces RCT assay was performed according to the methods initially described by Zhang et al. (30) with minor modification (31). CETP tg LDLr^−/−^ mice were treated with saline, a control ASO (15 mg/kg/wk), CETP ASO (15 mg/kg/wk), or anacetrapib (50 mg/kg/day). After two weeks of treatment, mice were administered ^3^H-cholesterol-labeled macrophages via intraperitoneal injection (approximately 5.25 mil dpm/7.5 mil cells/mouse). The mice were singly housed in wire bottom cages for 72 h, plasma samples were taken at 24 and 48 h, and feces were collected over the entire 72 h period. After 72 h, the mice were euthanized, and a terminal plasma sample was collected along with a liver sample. A 20 µl aliquot of plasma from each time point/mouse was counted for dpm by liquid scintillation counting (LSC). Liver tissue was extracted according to previously described methods (32), and the isolated lipid extracts were dried under N_2_. The dried extract was resuspended in scintillation cocktail and counted by LSC. Finally, the amount of radiolabeled fecal cholesterol and bile acid was evaluated according to previously published methods (31).

### Ex vivo radiolabeled HDL-CEth exchange study

The relative ability of the CETP ASO and anacetrapib to inhibit neutral lipid exchange ex vivo was evaluated by the following method. Plasma samples were collected from CETP tg LDLr^−/−^ mice treated with either saline, control ASO (15 mg/kg/wk), CETP ASO (15 mg/kg/wk), or anacetrapib (50 mg/kg/day) for three weeks. Lipid exchange was assayed by incubating 5 µl of plasma with 20,000 dpm of radiolabeled human HDL_3_, and total volume was brought up to 100 µl with the addition of 1 mM of the LCAT inhibitor, 5,5-dithiobis-(2-nitorbenzoic acid) (DTNB) in PBS. The samples were incubated at 37°C for 0.5, 3, and 12 h, and then apoB-bound lipoproteins were precipitated by the addition of 100 µl of phosphotungstate and magnesium salt. After centrifugation, 50 μl of the supernatant was collected and counted by LSC.

### Evaluation of TG secretion

The effect of CETP inhibition on TG secretion was evaluated in CETP tg LDLr^−/−^ mice treated with either saline, control ASO (15 mg/kg/wk), CETP ASO (15 mg/kg/wk), or anacetrapib (50 mg/kg/day) for four weeks. After baseline plasma samples were collected, mice were injected with the detergent poloxamer 407 according to previously described protocols (33). Subsequent plasma samples were collected at 1, 3, and 6 h after injection, and plasma TG was measured by enzymatic assay. The results are reported as the percentage increase from baseline plasma sample.

### Liver lipid analysis

Liver TG concentration was assayed in the CETP tg LDLr^−/−^ mice according to previously described protocols (34).

### Assessment of aortic cholesterol content

Western diet-fed CETP tg LDLr^−/−^ mice were treated with either saline, control ASO (15 mg/kg/wk), CETP ASO (15 mg/kg/wk), or anacetrapib (50 mg/kg/day) for 12 weeks. During the first 4 weeks of treatment, mice were injected with ASO or saline vehicle weekly and thereafter treated biweekly for the remainder of the study. After 12 weeks of treatment, the animals were euthanized, and a terminal plasma sample was taken along with liver tissue. Aortas were perfused with buffered saline and stored in 10% neutral buffered formalin. The aortas were stripped of any remaining fat and connective tissue and analyzed for total and FC content by GC analysis according to methods described previously (35). Aortic CE was calculated by TC – FC × 1.67, and protein was quantified by Lowry assay.

### Statistical analysis

All values are expressed as mean ± SEM. To determine statistical significance, one way ANOVA analysis with Tukey's post-hoc test was carried out using GraphPad Prism 5™ software with statistical significance being set at *p*<0.05.

## RESULTS

To compare and contrast the effects of a CETP ASO and SMI on target expression and enzymatic activity as well as their impact on plasma lipids and lipoproteins, dose-response experiments were performed. These studies were conducted in the CETP tg and CETP tg LDLr^−/−^ mice, two models with distinctly different lipoprotein profiles. In the CETP tg mice, the majority of cholesterol is carried in HDL particles, which allows study of how the inhibitors affect hepatic CETP expression and plasma protein levels in a model where sterol feedback through the LDL receptor is maintained. Conversely, in the CETP tg LDLr^−/−^ mice, the bulk of the cholesterol circulates in VLDL and LDL. In these mice, RCT via LDL through the LDL receptor is blocked; therefore, this model affords more direct comparison of how the increases in HDL facilitated by either CETP inhibitor affects HDL functionality, HDL-mediated RCT, and finally aortic cholesterol accumulation.

After six weeks, CETP tg mice treated with either the CETP ASO or anacetrapib displayed dose-dependent increases in plasma cholesterol, primarily due to increases in HDL-C (**Table 1**). Administration of the CETP ASO at 15 mg/kg/wk significantly increased plasma cholesterol and HDL-C by 34% and 41%, respectively, when compared with saline controls. Similarly, administration of anacetrapib at 100 mg/kg/day resulted in a nonsignificant 24% increase in plasma cholesterol and a significant 33% increase in HDL-C when compared with saline controls. Predictably, when compared with saline controls, mice treated with the CETP ASO displayed significant, dose-dependent reductions in CETP mRNA and protein levels across all of the treatment groups, reaching maximal reductions of 95% and 97%, respectively, at the highest administered dose. Conversely, anacetrapib tended to increase CETP mRNA expression and significantly elevated CETP protein levels in a dose-dependent manner when compared with the saline group, with the highest dose of anacetrapib increasing CETP protein level by 82%. Despite increasing CETP protein, high-dose anacetrapib treatment significantly reduced CETP activity by 25% when compared with saline controls. When the two inhibitors were directly evaluated, the CETP ASO at 15 mg/kg/wk significantly lowered activity compared with all anacetrapib-treated mice.

**TABLE 1.** Effect of CETP inhibition on plasma lipids, CETP mRNA, protein, and activity in CETP tg mice

Treatment Group	TPC (mg/dl)	TG (mg/dl)	HDL-C (mg/dl)	CETP mRNA (% Saline)	CETP Protein (µg/ml)	CETP Activity (% Saline)
Saline	162 ± 8	79 ± 7	116 ± 7	100 ± 6	17 ± 2	100 ± 2
Control ASO (15 mg/kg/wk)	180 ± 10	88 ± 6	124 ± 9	127 ± 27	19 ± 2	99 ± 11
CETP ASO (15 mg/kg/wk)	217 ± 8*	85 ± 5	164 ± 7^†^	5 ± 1^	3 ± 0.4^	24 ± 1^†^
CETP ASO (5 mg/kg/wk)	202 ± 2*	85 ± 6	151 ± 3*	13 ± 2^	5 ± 0.6^	42 ± 28^†^
CETP ASO (1.5 mg/kg/wk)	180 ± 7	91 ± 4	132 ± 7	41 ± 8^	9 ± 0.5^	68 ± 1^†^
Anacetrapib (100 mg/kg/day)	201 ± 8*	67 ± 2	154 ± 6*	132 ± 8	31 ± 0.4*	78 ± 4^†^
Anacetrapib (50 mg/kg/day)	176 ± 6^#^	75 ± 5	135 ± 5	133 ± 12	27 ± 3*	75 ± 3^†^
Anacetrapib (10 mg/kg/day)	177 ± 6^#^	75 ± 5	137 ± 5	108 ± 8	24 ± 2*	87 ± 5

Values represent mean ± SEM, n = 4–6/group after six weeks of treatment. *P < 0.05 compared with saline; ^†^*P* < 0.05 compared with saline and control ASO; ^#^*P* < 0.05 compared with CETP ASO (15 mg/kg/wk); ^*P* < 0.05 compared with saline, control ASO, and all anacetrapib groups.

When the two CETP drugs were compared in the hyperlipidemic CETP tg LDLr^−/−^ mice, a broader effect of CETP inhibition on lipoprotein metabolism was revealed (**Table 2**). After six weeks of treatment, both compounds reduced total plasma cholesterol in a dose-dependent manner. The cohort of CETP tg LDLr^−/−^ mice given the highest dose of the CETP ASO (15 mg/kg/wk) displayed a 38% reduction in plasma cholesterol when compared with the saline group. A similar decrease (41% reduction) was observed in mice treated with anacetrapib at 50 mg/kg/day. Despite these significant reductions in total plasma cholesterol, mice administered either CETP inhibitor displayed significant increases in HDL-C. For example, animals treated with the highest dose of each drug displayed a significant 8-fold increase in HDL-C. The effects on HDL-C were not dose-responsive, with the lowest doses of either the ASO or anacetrapib providing comparable increases. These results suggest that the reductions in CETP activity at the lower doses were sufficient to raise HDL-C; however, to observe a positive effect on the predominant lipoprotein subclasses (i.e., VLDL and LDL), additional drug was required. Due to the lack of a functional LDL receptor, it is important to note that this effect of CETP inhibition on reducing VLDL and LDL could be model specific. Currently we speculate the reductions in total cholesterol observed in the CETP tg LDLr^−/−^ following treatment with higher doses of the CETP inhibitors could be due to inhibiting the transfer of CE from HDL and blocking the futile exchange of lipid between apoB-bound lipoproteins, perhaps allowing for their gradual removal by less efficient receptors, such as scavenger receptor B1 (SR-B1) and LDL receptor-related protein (LRP) (36, 37).

**TABLE 2.** Effect of CETP inhibition on plasma lipids, CETP mRNA, protein, and activity in CETP tg LDLr^−/−^ mice

Treatment Group	TPC (mg/dl)	TG (mg/dl)	HDL-C (mg/dl)	CETP mRNA (% Saline)	CETP Protein (µg/ml)	CETP Activity (% Saline)
Saline	2286 ± 123	670 ± 75	10 ± 2	100 ± 6	46 ± 2	100 ± 2
Control ASO (15 mg/kg/wk)	2475 ± 179	603 ± 84	16 ± 6	92 ± 7	43 ± 4	90 ± 5
CETP ASO (15 mg/kg/wk)	1420 ± 134*^,‡^	194 ± 27*^,‡^	80 ± 4*^,‡^	11 ± 1^	10 ± 1^	19 ± 1*^,‡^
CETP ASO (5 mg/kg/wk)	1801 ± 125^‡^	257 ± 17*^,‡^	78 ± 4*^,‡^	19 ± 4^	12 ± 0.4^	27 ± 1*^,‡^
CETP ASO (1.5 mg/kg/wk)	1853 ± 189	268 ± 32*^,‡^	86 ± 4*^,‡^	25 ± 2^	22 ± 3^	35 ± 2*^,‡^
Anacetrapib (100 mg/kg/day)	1480 ± 152*^,‡^	653 ± 115	81 ± 7*^,‡^	99 ± 15	45 ± 4	14 ± 1*^,‡^
Anacetrapib (50 mg/kg/day)	1359 ± 116*^,‡^	553 ± 95	79 ± 6*^,‡^	98 ± 8	49 ± 5	14 ± 0.4*^,‡^
Anacetrapib (10 mg/kg/day)	1565 ± 160*^,‡^	544 ± 18	84 ± 7*^,‡^	96 ± 17	41 ± 6	21 ± 1*^,‡^
Anacetrapib (2 mg/kg/day)	1807 ± 76^‡^	576 ± 18	86 ± 2*^,‡^	83 ± 7	45 ± 3	26 ± 1*^,‡^

Values represent mean ± SEM, n ≥ 4/group after six weeks of treatment. *P < 0.05 compared with saline; ^‡^*P* < 0.05 compared with control ASO; ^*P* < 0.05 compared with saline, control ASO, and all anacetrapib groups.

Interestingly, after 6 weeks of treatment, CETP tg LDLr^−/−^ mice given the CETP ASO had significant and dose-responsive reductions in plasma TG (Table 2). Mice administered the ASO at the highest dose displayed a 69% reduction in plasma TG, an effect that was not observed in the control ASO administered group. LDLr^−/−^ mice on a similar diet and dosed with either the CETP or control ASO (data not shown) displayed no change in plasma TG levels suggesting that this effect was dependent upon the presence of CETP. Since CETP tg LDLr^−/−^ mice treated with anacetrapib did not show an effect on plasma TG after 6 weeks of treatment, additional assays were conducted to see if the CETP ASO altered TG secretion or hepatic TG level. As shown in **Table 3**, compared with the saline and control ASO groups, treatment with either CETP inhibitor displayed a reduction in TG secretion following administration of a detergent to block VLDL-TG catabolism. Additionally, while there was a trend for an increase in liver TG in mice treated with the CETP inhibitors compared with the control ASO group, neither inhibitor had a significant effect on liver TG. These initial studies suggest both the CETP ASO and anacetrapib can alter TG secretion; however, the mechanism accounting for the differential in plasma TG between the two inhibitors remains to be elucidated.

**TABLE 3.** Effect of CETP inhibition on TG secretion and liver TG

	Time After Detergent Injection (% increase from baseline)			
Treatment Group	1 h	3 h	6 h	Liver TG (μg/mg)
Saline	97 ± 5	289 ± 12	353 ± 9	65 ± 6
Control ASO (15 mg/kg/wk)	115 ± 7	323 ± 16	398 ± 8	39 ± 9
CETP ASO (15 mg/kg/wk)	43 ± 4*^,‡^	198 ± 16*^,‡^	323 ± 13^‡^	71 ± 22
Anacetrapib (50 mg/kg/day)	53 ± 3*^,‡^	180 ± 13*^,‡^	279 ± 25*^,‡^	79 ± 19

Values represent mean ± SEM, n = 4–5/group after six weeks of treatment. *P < 0.05 compared with saline; ^‡^*P* < 0.05 compared with control ASO.

**Fig. 1.**
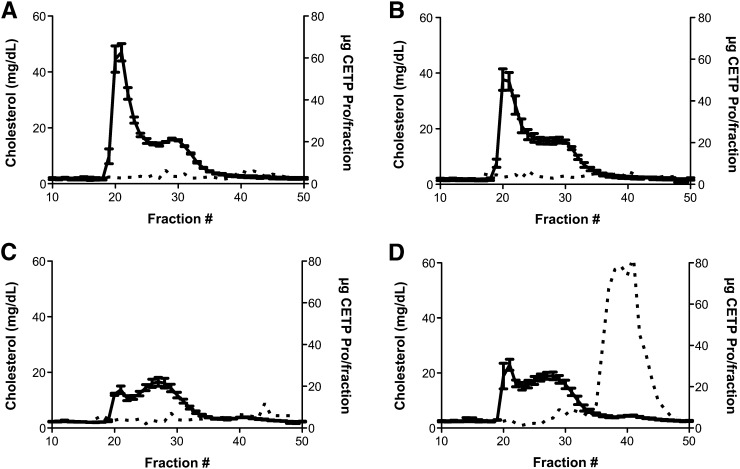
Effects of CETP inhibition on lipoprotein cholesterol and CETP protein distribution in CETP tg LDLr^−/−^ mice. Lipoprotein cholesterol (solid line) and CETP protein (dotted line) distribution in (A) saline, (B) control ASO, (C) CETP ASO, and (D) anacetrapib groups after 12 weeks of treatment. Lipoprotein cholesterol distribution curves for each treatment group are derived from three pooled plasma samples from three mice. HPLC fractions were combined, and the distribution of CETP protein across the lipoprotein subclasses for each treatment group was determined by ELISA.

Similar to our previous observations in the CETP tg mice, CETP tg LDLr^−/−^ mice administered the CETP ASO displayed significant reductions in CETP mRNA, protein, and activity level, with the highest dose of 15 mg/kg/wk displaying 89%, 90%, and 81% reductions, respectively, relative to the saline group (Table 2). In contrast, anacetrapib did not affect CETP mRNA or protein levels. Interestingly, in this model, anacetrapib had a more potent effect on CETP activity, and at the highest doses, activity was suppressed by 86% compared with the saline group. As shown in **Fig. 1**, analysis of HPLC fractions from CETP tg LDLr^−/−^ mice treated with anacetrapib indicates the majority of CETP protein is found in CETP-HDL complexes; however, additional CETP protein was also associated with LDL. This additive effect on lowering CETP activity across all the anacetrapib groups suggests that the higher concentration of LDL in the hyperlipidemic CETP tg LDLr^−/−^ mice provides more substrate for anacetrapib to bind CETP, effectively reducing the amount of protein available to transfer lipid in the activity assay. Due to a plateau in the pharmacology of anacetrapib at the 100 mg/kg/day dose in the CETP tg LDLr^−/−^ mice, 50 mg/kg/day was selected for use in all subsequent studies.

**Fig. 2.**
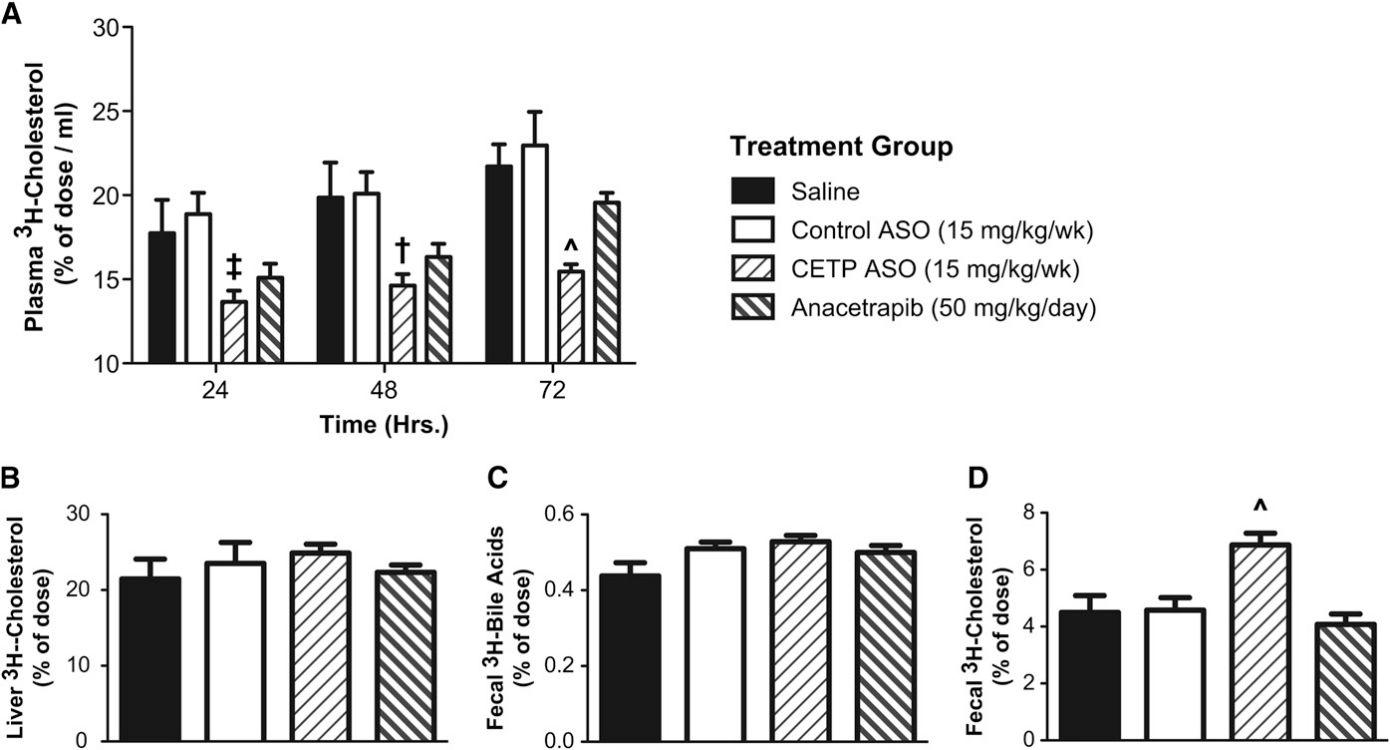
Effect of CETP inhibitors on RCT in CETP tg LDLr^−/−^ mice. (A) Clearance of ^3^H-cholesterol from plasma over a 72 h period in mice treated with either saline, control ASO, CETP ASO, or anacetrapib for two weeks and IP injected with radiolabeled macrophages. Amount of ^3^H-cholesterol in (B) liver, (C) fecal bile acids, and (D) feces (n = 6 for saline and control groups, n = 10 for CETP ASO and anacetrapib groups). ^*P* < 0.05 compared with saline, control ASO, and anacetrapib groups; ^‡^*P* < 0.05 compared with control ASO; ^†^*P* < 0.05 compared with saline and control ASO groups.

A key antiatherogenic function of HDL is to facilitate RCT, the delivery of excess cholesterol from the periphery back to the liver where it can be eliminated as bile acid and biliary cholesterol. In vivo RCT analysis was carried out in CETP tg LDLr^−/−^ mice treated with either the CETP ASO or anacetrapib in order to assess the functional impact the increases in HDL achieved with the two types of CETP inhibitors. As shown in **Fig. 2**, the ASO and SMI had significantly different effects on RCT. Fig. 2A demonstrates that in the saline and control ASO groups, where CETP activity was maintained and HDL-CE was transferred to apoB-bound lipoproteins, the radiolabeled cholesterol accumulated in plasma. Conversely, when CETP was inhibited, the level of radiolabeled cholesterol in plasma was reduced, suggesting enhanced uptake. This effect was most prominent in the CETP ASO group, where after 72 h, there was significantly less plasma radiolabel when compared with all other treatment groups, including anacetrapib. No significant effect of either CETP drug on the accumulation of radiolabel in the liver or fecal bile acids could be detected (Fig. 2B, C); however, mice treated with the CETP ASO displayed a significant 53% increase in fecal ^3^H-cholesterol when compared with the saline, control ASO, and anacetrapib groups (Fig. 2D). Thus, the reduced accumulation in plasma ^3^H-cholesterol and increased elimination of ^3^H-cholesterol in the feces demonstrated enhanced RCT in mice treated with the CETP ASO.

**Fig. 3.**
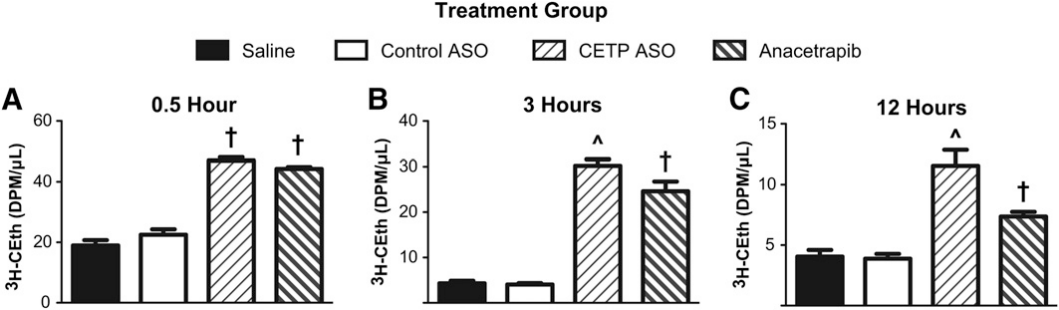
Effect of CETP inhibition on ex vivo lipid exchange. Plasma samples from CETP tg LDLr^−/−^ mice treated with either saline, control ASO, CETP ASO, or anacetrapib (n = 6/group) were incubated with radiolabeled HDL for (A) 0.5 h, (B) 3 h, and (C) 12 h. ApoB-bound lipoproteins were precipitated, and the amount of radiolabel remaining in the supernatant was measured by LSC. ^*P* < 0.05 compared with saline, control ASO, and anacetrapib groups; ^†^*P* < 0.05 compared with saline and control ASO groups.

Analyses of plasma lipids and lipoproteins on terminal plasma samples from the macrophage RCT assay were performed to detect any potential differences that might account for the improvements in RCT observed in mice treated with the ASO relative to anacetrapib (**Table 4**). Similar to what was observed in the dose-response studies in CETP tg LDLr^−/−^ mice, treatment with the CETP ASO was associated with reductions in total plasma cholesterol and TG (45% and 62%, respectively, compared with the saline group). With the benefit of the additional mice used in the RCT assay, greater statistical power was achieved, and the reductions in total plasma cholesterol were significant compared with the saline, control ASO, and anacetrapib groups. Mice administered anacetrapib in this study displayed a significant reduction in total plasma cholesterol of 25% when compared with the control groups; however, no change in plasma TG could be detected. Interestingly, both CETP inhibitors provided similar significant increases in HDL-C and the amount of radiolabel associated with HDL particles; however, only mice treated with the ASO displayed increased HDL apoA-I. To further investigate any other differences between the treatment groups, HDL from mice treated with the ASO or SMI for six weeks were isolated by ultracentrifugation and analyzed for lipid and protein content. No additional differences in lipid composition or overall protein mass were observed between mice treated with the ASO and mice treated with the SMI (**Table 5**). Alternatively, to evaluate whether differences in apoB-bound lipoproteins could account for the differential effects on RCT, the relative ability of either inhibitor to block lipid exchange ex vivo was examined. As shown in **Fig. 3**, plasma samples from mice treated with the CETP ASO were more effective in inhibiting the exchange of radiolabeled cholesteryl ether from donor HDL to native apoB-bound lipoproteins over a 12 h period. These results suggest that the additional reductions in total cholesterol and TG in mice treated with the ASO could limit substrate available for exchange and provide a secondary means to inhibit CETP activity, and along with the enhanced association of apoA-I on HDL, might potentially account for the enhanced RCT.

**TABLE 4.** Macrophage RCT assay post-injection plasma data

Treatment Group	TPC (mg/dl)	TG (mg/dl)	HDL-C (mg/dl)	HDL/apoA-I (mg/dl)	WP ^3^H-Cholesterol (DPM/20 µl)	HDL ^3^H-Cholesterol (DPM/20 µl)
Saline	1883 ± 182	553 ± 121	19 ± 6	20 ± 1	22790 ± 1390	177 ± 23
Control ASO (15 mg/kg/wk)	1701 ± 132	485 ± 88	19 ± 7	21 ± 2	24103 ± 2100	189 ± 26
CETP ASO (15 mg/kg/wk)	1042 ± 31^	212 ± 13^	63 ± 4^†^	47 ± 6^	16224 ± 456^	650 ± 41^†^
Anacetrapib (50 mg/kg/day)	1404 ± 50^†^	631 ± 22	63 ± 4^†^	28 ± 3	20533 ± 606	687 ± 23^†^

Values represent mean ± SEM, n = 6 for saline and control groups, n = 10 CETP ASO and anacetrapib groups. ^*P* < 0.05 compared with saline, control ASO, and anacetrapib groups. ^†^*P* < 0.05 compared with saline and control ASO. WP, whole plasma.

**TABLE 5.** Effect of CETP inhibition on HDL composition

Treatment Group	FC %	PL %	Pro %	CE %	TG %
Saline	8.2	24.3	39.4	11.5	16.6
Control ASO (15 mg/kg/wk)	5.0	20.1	37.8	14.7	22.4
CETP ASO (15 mg/kg/wk)	4.0	31.8	39.3	21.7	3.1
Anacetrapib (50 mg/kg/wk)	4.7	33.1	39.9	20.4	1.9

Values represent the percentage of total mass for each component.

The primary objective of any HDL therapeutic is to improve CVD outcomes. Accumulation of excess cholesterol in the arterial intima is a fundamental step in atherosclerotic plaque progression (38). Therefore, the ability of the CETP ASO and anacetrapib to mitigate aortic cholesterol deposition was evaluated in the CETP tg LDLr^−/−^ mice over a 12-week period (**Table 6**). The effects of both drugs on plasma lipids, lipoproteins, and CETP were similar to that observed in the 6-week dose-response study. Compared with the saline group, both the CETP ASO and anacetrapib provided significant reductions in total plasma cholesterol of 47% and 39%, with concomitant 16- and 15-fold increases in HDL-C, respectively. Mice administered the CETP ASO also displayed a significant reduction in plasma TG of 72%. The lowering of plasma cholesterol observed with both inhibitors was primarily due to reductions in VLDL-C (Fig. 1); however, this effect was greatest in mice treated with the ASO, where greater reductions in LDL-C were also observed.

**TABLE 6.** Aortic cholesterol study 12-week data

Treatment Group	TPC (mg/dl)	TG (mg/dl)	HDL-C (mg/dl)	CETP mRNA (% Saline)	CETP Protein (µg/ml)	CETP Activity (% Saline)	Aortic FC (µg/mg Pro)	Aortic CE (µg/mg Pro)
Saline	2662 ± 99	594 ± 49	5 ± 2	100 ± 8	94 ± 5	100 ± 3	43 ± 3	95 ± 9
Control ASO (15 mg/kg/wk)	2575 ± 190	589 ± 60	7 ± 5	91 ± 9	86 ± 6	96 ± 4	39 ± 2	77 ± 5
CETP ASO (15 mg/kg/wk)	1422 ± 56^†^	168 ± 12^	78 ± 2^†^	9 ± 1^	19 ± 1^	20 ± 1^†^	35 ± 2	67 ± 7*
Anacetrapib (50 mg/kg/day)	1634 ± 58^†^	508 ± 28	73 ± 4^†^	90 ± 6	128 ± 4^†^	12 ± 1^†^	42 ± 2	73 ± 7

Values represent mean ± SEM, n ≥ 11/group. ^†^*P* < 0.05 compared with saline and control ASO; ^*P* < 0.05 compared with saline, control ASO, and anacetrapib groups; *P < 0.05 compared with saline.

**Fig. 4.**
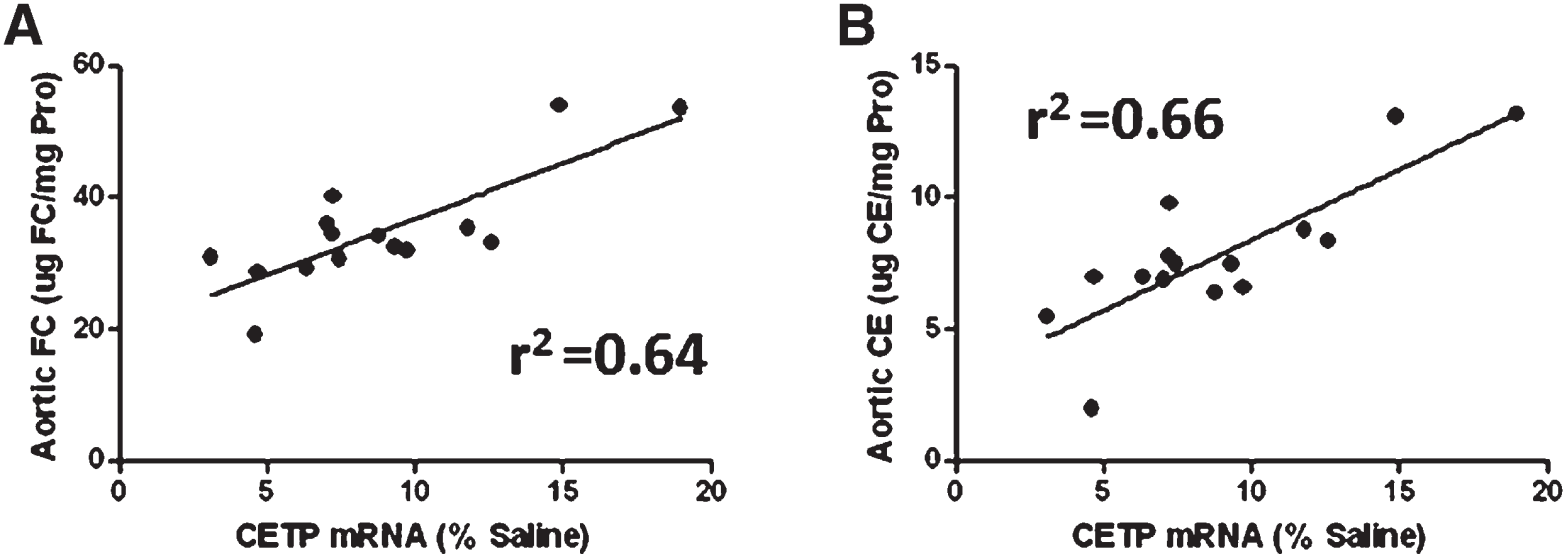
CETP mRNA level is predictive of aortic cholesterol in CETP tg LDLr^−/−^ mice treated with a CETP ASO. Relationship between CETP mRNA and (A) aortic FC and (B) aortic CE in mice (n = 15) administered CETP ASO for 12 weeks.

The proposed antiatherogenic mechanism of CETP inhibition involves blockade of neutral lipid exchange between apoB-bound lipoproteins and HDL, which should attenuate the atherogenic potential of VLDL and LDL and enhance HDL-mediated RCT. Therefore inhibition of CETP would be predicted to reduce the amount of VLDL and LDL cholesterol available for vascular deposition and to accelerate the removal of excess plaque cholesterol by HDL. To confirm these expectations, aortic FC and CE content was measured in CETP-inhibited mice. Of all the groups evaluated, mice treated with the CETP ASO displayed the greatest reductions in aortic CE and FC at 30% and 19%, respectively, when compared with the saline group (Table 6). However, only reductions in aortic CE were significant when compared with the saline group, and no other significant differences could be established between the CETP ASO and other treatment groups. Since the use of pharmacological inhibitors will result in a range of effects on target inhibition, the relationship between ASO-mediated reduction of CETP mRNA and aortic CE and FC content was correlated. As shown in **Fig. 4**, CETP mRNA level was positively correlated with both aortic CE and FC, indicating the greater the reductions in CETP mRNA with ASO treatment could result in more positive effects on aortic cholesterol accumulation.

## DISCUSSION

The primary goal of these studies was to characterize and compare the efficacy of an ASO targeting CETP in reducing hepatic mRNA, protein, and enzymatic activity to the potent CETP SMI anacetrapib. Once an optimal dose of CETP ASO was determined, further studies explored the effects on plasma lipids, HDL-C, RCT, and aortic cholesterol accumulation. In the two tg models tested, the CETP ASO and anacetrapib increased HDL-C to comparable levels. In hyperlipidemic CETP tg LDLr^−/−^ mice, both inhibitors provided reductions in total plasma cholesterol and CETP activity. Uniquely, CETP tg LDLr^−/−^ mice administered the ASO displayed positive effects on RCT, decreases in plasma TG, and reductions in CETP mRNA that were associated with reduction in aortic cholesterol.

It is important to note the pharmacological profile observed in tg mice may be difficult to extrapolate to observations in man. For example, in CETP tg and CETP tg LDLr^−/−^ mice, which have levels of CETP plasma protein of at 19 and 46 μg/ml, respectively, have 5- to 8-fold higher plasma levels than that observed in humans, which is typically 1.8 μg/ml (39). Given that SMIs such as anacetrapib are thought to inactivate CETP by binding the protein to the HDL particle (22), the enhanced association of CETP with HDL demonstrated in Fig. 1 could restrict HDL functionality and may account for some of the differential effects observed between the two inhibitors. Like most tg mouse models, these mice may provide an exaggerated view of the role of CETP in lipoprotein metabolism, and when combined with LDLr deficiency, they may further accentuate apparent phenotypic changes due to the severe level of hyperlipidemia induced in the model. With these limitations in mind, the use of tg mice enabled the development of ASOs targeting human CETP mRNA. Furthermore, the subsequent comparative studies provided novel insights into the distinct mechanisms of action of the two classes of drugs and the impetus to perform further studies in more relevant preclinical models.

The dose-response studies performed in the CETP tg and CETP tg LDLr^−/−^ mice highlighted the differences between the CETP ASO and anacetrapib. In both models, the CETP ASO provided a dose-linear and consistent reduction in CETP mRNA with concordant decreases in protein and activity. In the CETP tg mice, treatment with anacetrapib resulted in comparable increases in HDL-C to that observed in the ASO groups. However, the SMI provided only modest reductions in CETP activity. Further, the elevations in HDL-C in anacetrapib-treated CETP tg mice appeared related to increases in CETP protein. Similar dose-dependent relationships between CETP protein and HDL-C have been observed in clinical evaluations of CETP SMIs (17, 40) and are most likely due to the formation of CETP-HDL complexes. The CETP activity assay used in these studies measures the exchange of a fluorescent probe from exogenous donor and acceptor particles; therefore, the attenuated reduction in activity could be due to free CETP present in plasma isolates. In the hyperlipidemic CETP LDLr^−/−^ mice, anacetrapib and the CETP ASO produced comparable increases in HDL-C and similar reductions in CETP activity. HPLC analysis on plasma samples from CETP tg LDLr^−/−^ mice treated with anacetrapib found that the majority of CETP was associated with HDL; however, the protein was also found in fractions containing the apoB-bound lipoproteins, primarily LDL. These results suggest that, under certain conditions in which apoB-bound lipoproteins are elevated, anacetrapib can engage these additional particles to enhance CETP sequestration and inhibition. However, despite the fundamental differences described above, these initial comparative studies revealed that both drug classes can achieve comparable increases in HDL-C and reductions in CETP enzymatic activity.

A surprising finding from our comparison studies was that treatment with the ASO enhanced RCT, whereas anacetrapib did not. This result was unexpected, considering that in the CETP tg LDLr^−/−^ mice, both inhibitors provided comparable reductions in CETP activity and increases in HDL-C. Additional analyses suggest the improvement in RCT in mice treated with the ASO compared with the SMI could be related to increases in HDL apoA-I and reductions in plasma TG. Similar in vivo RCT experiments conducted in human A-I tg mice found treatment with agents that increased apoA-I expression and production rate resulted in increased RCT (41). Furthermore, while multiple preclinical experiments have shown CETP SMIs have a favorable result on RCT (42, 43), differential effects of anacetrapib on RCT have been reported. Two independent evaluations of anacetrapib in hamsters found the type of diet used in the study could have a major impact on the effectiveness of the drug. For example, in one study conducted in chow-fed hamsters, anacetrapib had no effect on RCT (44). Conversely, in a separate study, when hamsters were maintained on a high-fat diet, anacetrapib increased RCT, resulting in an increase in radiolabeled cholesterol and bile acid in the feces (45). The authors cite that the use of a high-fat diet increased plasma cholesterol and TG, resulting in a dyslipidemia more relevant to what would be observed in the clinic. Interestingly, in the RCT study conducted with the dyslipidemic hamsters, treatment with anacetrapib also resulted in a significant reduction in plasma TG, a finding that is similar to what we observed with the CETP ASO. Potentially in a dyslipidemic state, reductions in plasma TG would limit the amount of TG available for exchange for HDL-CE and provide an additional mechanism for inhibiting CETP. This additional effect on CETP activity could result in a more efficient delivery of HDL-CE to the liver and improve RCT. While these preliminary observations require additional experimentation, they do suggest that the efficacy of CETP inhibitors could be limited in the background of hypertriglyceridemia.

The mechanism by which the CETP ASO lowers plasma TG in the hyperlipidemic tg mice is not fully understood. The ability of the CETP ASO to lower plasma TG is consistent with observations made in other tg mouse studies (46) and in patients with CETP loss of function mutations (47). Conversely, relationships between CETP and plasma TG have been described in patients with type 2 diabetes, in which CETP protein and activity were positively associated with plasma TG (48). In clinical trials, inhibition of CETP with SMIs significantly lowered plasma TG (17), and administration of torcetrapib in patients with combined dyslipidemias displayed enhanced postprandial VLDL-TG metabolism (49). In CETP tg LDLr^−/−^ mice, treatment with anacetrapib initially lowered plasma TG; however, this reduction was transient (data not shown). Preliminary studies have found that the differences in plasma TG between the ASO and anacetrapib were not due to increase in hepatic TG accumulation or a reduction in hepatic TG secretion. Interestingly, mice treated with either inhibitor displayed impaired TG secretion after TG hydrolysis was inhibited by the injection of the detergent poloxamer 407. These results support observations from the clinic, where torcetrapib monotherapy lowered plasma TG and reduced the production rate of apoB-48 (50). Given that mice secrete both apoB-100- and apoB-48-bound lipoproteins from the liver and intestine, the observed delay in TG secretion in mice treated with the CETP inhibitors could indicate an effect on apoB-48 production. These initial observations suggest that the relationship between inhibition of CETP and plasma TG with either an ASO or SMI is highly complex, and the effects of CETP inhibition on TG and apoB metabolism will be the focus of a future article.

Atherosclerotic plaque formation is a complex process that is primarily facilitated by two aspects: the accumulation of excess cholesterol in the arterial intima and the subsequent immune response resulting in the recruitment of monocytes/macrophages (38). To examine whether the improvements in RCT and reductions in plasma cholesterol observed in mice treated with the CETP ASO had a beneficial effect on deposition of cholesterol in the aorta, CETP tg LDLr^−/−^ mice were treated with the ASO for 12 weeks. Compared with the other treatment groups, mice administered the CETP ASO had the greatest reduction in aortic CE and FC; however, the reduction in aortic CE was only significant when compared with the saline group and statistically significant differences could not be detected between the control ASO and anacetrapib groups. Unfortunately, without a significant difference when compared with both control groups, it must be concluded neither inhibitor had an effect on aortic cholesterol. This lack of a potent effect on aortic cholesterol may have been due to a combination of the highly elevated levels of proatherogenic lipoproteins in the tg mice and the inherent variation in target reduction produced with the use of pharmacologic agents. Therefore, to further elucidate the relationship between CETP and aortic cholesterol, the ASO-mediated reductions in CETP mRNA were compared with the levels of aortic CE and FC. The resulting analysis found a strong positive correlation (Fig. 4A, B). Within the CETP ASO group, mice that had the greatest reduction in CETP mRNA also displayed the greatest reduction in aortic cholesterol content. These associations between CETP and plaque progression have also been observed in atherosclerosis studies conducted in nonhuman primates, in which the CETP protein was predictive of coronary artery intimal area (51). Furthermore, these results are consistent with other atherosclerosis studies in tg mice, in which CETP expression was proatherogenic (46, 52, 53), and in cholesterol-fed rabbits, in which administration of a CETP ASO also reduced aortic cholesterol (54). The inability of anacetrapib to significantly reduce aortic cholesterol is most likely model dependent, since both torcetrapib and dalcetrapib significantly reduced plague burden in atherosclerosis models that endogenously express CETP (55, 56).

In summary, despite the failure of torcetrapib and dalcetrapib, the continued development of safe and potent CETP SMIs such as anacetrapib and evacetrapib demonstrates that CETP inhibition still holds promise as a potential therapeutic strategy. Our studies revealed that inhibition of CETP with an ASO and anacetrapib similarly reduced CETP activity and increased HDL-C levels. Additionally, in a preclinical tg model with profound hyperlipidemia, administration of the CETP ASO demonstrated that significant reductions in plasma TG, positive effects on macrophage RCT, and reductions in CETP mRNA were associated with less accumulation of aortic cholesterol. These results were validated by recent findings from the Copenhagen City Heart Study, in which genetic inhibition of CETP was associated with an increase in HDL-C and decreases in plasma TG, LDL-C, non-HDL-C, and adverse events (47). Given its unique mechanism of action, an ASO therapeutic agent might more faithfully recapitulate the CETP genetic loss-of-function phenotype and thus provide beneficial effects on CVD outcomes in man.
